# Perforation of the Ileum

**Published:** 1845-02

**Authors:** 


*Perforation of the ileum.*—Dr. Favell, at a meeting of the Sheffield Medical Society, exhibited a portion of intestine taken from the body of a young lady, aged 18 years, who died a few days previously of severe and extensive peritonitis induced by perforation of the ileum. The patient had been in delicate health for a considerable time, and had suffered from three attacks of low fever within a comparatively short period. She appeared to be gradually recovering from the last attack, when, on the 23d of October, she was suddenly seized with severe abdominal pain, sickness, and other symptoms of peritoneal inflammation, and died, after much suffering, during the night of the 26th. On examining the body after death, the whole of the peritoneal covering of the intestines was found to be intensely injected, and the convolutions adhering by coagulable lymph. The pelvis contained a small quantity of fluid fæces; large patches of deep ulceration were observed in the lining membrane of the ileum and cæcum, and two perforations, each about the size of a split pea, were noticed in the former intestine, about six inches from its junction with the latter. The omentum was in a state of sphacellus—*Ibid.*

